# Recurrent laryngeal nerve palsy complicating subclavian line insertion: a case report

**DOI:** 10.4076/1752-1947-3-9034

**Published:** 2009-09-08

**Authors:** Jonathan M Fishman

**Affiliations:** 1 Department of Otolaryngology, The John Radcliffe Hospital, University of Oxford, Oxford, OX3 9DU, UK

## Abstract

**Introduction:**

Although recurrent laryngeal nerve injury has been described following central venous access via the jugular route, it has not previously been reported following access via the subclavian route.

**Case presentation:**

A 63-year-old man presented with acute dysphonia immediately after insertion of a Hickman line via the subclavian route. Flexible laryngoscopy revealed a left vocal fold palsy. A computed tomography scan from the skull base to the thoracic inlet showed no obvious abnormality other than an abducted left vocal cord.

The timing of the events and the computed tomography scan results strongly support the conclusion that the left recurrent laryngeal nerve was injured during insertion of the Hickman line, resulting in a left adductor vocal cord palsy.

**Conclusion:**

This case illustrates an unusual example of iatrogenic injury to the recurrent laryngeal nerve. It is important to recognize the possibility that such injuries may occur in order to prevent them.

## Introduction

Neurological injuries to the cervical plexus, brachial plexus, vagus, phrenic and recurrent laryngeal nerves are recognized complications of central venous line insertion. However, injury to the recurrent laryngeal nerve is presumably rare as it has not previously been reported following central venous access via the subclavian route [1].

## Case presentation

A 63-year-old Caucasian man presented with acute dysphonia immediately after insertion of a left-sided Hickman line under local anaesthetic, via the subclavian route. The subclavian line was inserted under ultrasound guidance and the procedure was without complications. He had undergone a general anaesthetic several days prior to the Hickman line being inserted, for debridement of an infected hip joint, and the presumed aetiology of the dysphonia was initially thought to be due to laryngeal trauma at the time of intubation. Since the voice failed to improve within the expected time, an otolaryngological opinion was requested.

**Figure 1:**
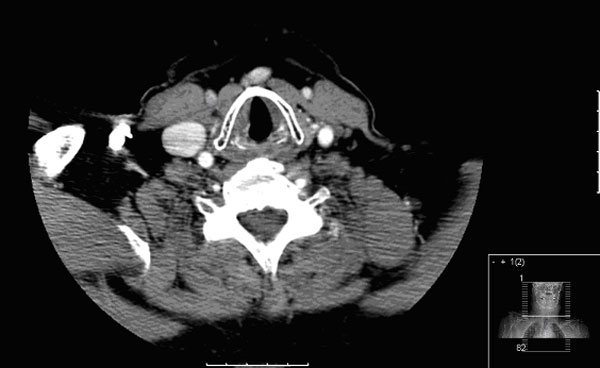
**Axial computed tomography through the vocal cords**. Computed tomography with contrast from the skull base to the superior mediastinum. No mass was demonstrated along the course of the vagus, or recurrent laryngeal nerve, in the neck, or in the superior mediastinum. Note the abducted left vocal fold in the above slice through the vocal cords.

Flexible fibreoptic laryngoscopy revealed an immobile left vocal cord, with the left vocal cord lying in a paramedian position. Palatal movements were normal thereby excluding the possibility of injury to the rest of the vagus nerve and confirming an isolated injury to the recurrent laryngeal nerve. A computed tomography (CT) scan from the skull base to the thoracic inlet showed no obvious abnormality other than an abducted left vocal cord (Figure 1).

## Discussion

This case report illustrates an unusual example of iatrogenic injury to the recurrent laryngeal nerve, an injury that is usually associated with thyroid, parathyroid, aortic, oesophageal or carotid endarterectomy surgery. The timing of the events and the CT scan results strongly support the conclusion that the left recurrent laryngeal nerve was injured during insertion of the Hickman line, resulting in a left adductor vocal cord palsy.

Evidence in favour of the Hickman line as the sole cause of the nerve palsy (as opposed to an idiopathic aetiology, an intubation-related injury, or any other cause) includes the following:

1.The timing of the events. The patient had a subclavian line inserted under local anaesthetic, several days after having been intubated for an unrelated operation. Insertion of the line was immediately followed by dysphonia.

2.The vocal cord palsy was unilateral and ipsilateral to the line insertion (left).

3.The voice failed to improve within the time expected for an intubation-related injury.

4.The CT scan excluded any other pathology from the skull base to the diaphragm.

Mechanisms of nerve injury may include one or more of the following, which need not be mutually exclusive [1]-[3]:

1.Direct trauma from needle cannulation, in view of the close anatomical relationship of the left recurrent laryngeal nerve with the left subclavian vessels.

2.Pressure neuropraxia, secondary to perineural haematoma.

3.Pressure neuropraxia, secondary to infection and inflammation of surrounding structures.

4.Surrounding thrombosis and fibrosis within and around the nerve.

In this case, direct trauma seems the most likely cause of nerve injury. When considering the treatment of such cases, it should be noted that recovery may take up to 12 months. Corrective surgery to the vocal fold should therefore be delayed for this period, allowing for conservative management of the injury in the interim [2,4].

## Conclusions

This case illustrates an unusual example of iatrogenic injury to the recurrent laryngeal nerve, after insertion of a Hickman line via the subclavian route. It is important to recognize the possibility that such injuries may occur in order to prevent them.

## Abbreviation

CT: computed tomography.

## Consent

Written informed consent was obtained from the patient for publication of this case report and any accompanying images. A copy of the written consent is available for review by the Editor-in-Chief of this journal.

## Competing interests

The authors declare that they have no competing interests.

## Authors' contributions

JMF was the primary researcher and writer of the manuscript.

## Disclosure

This case was presented at the Joint Annual Royal Society of Medicine/ENT-UK Meeting, Westminster, London, Autumn 2007.
